# Frequency and predictors of headache in the first 12 months after traumatic brain injury: results from CENTER-TBI

**DOI:** 10.1186/s10194-024-01751-0

**Published:** 2024-03-25

**Authors:** Emilie Isager Howe, Nada Andelic, Cathrine Brunborg, Marina Zeldovich, Eirik Helseth, Toril Skandsen, Alexander Olsen, Silje C. R. Fure, Alice Theadom, Katrin Rauen, Benedikte Å. Madsen, Bram Jacobs, Joukje van der Naalt, Maria Carmela Tartaglia, Cathrine Elisabeth Einarsen, Gøril Storvig, Erling Tronvik, Cathrine Tverdal, Nicole von Steinbüchel, Cecilie Røe, Torgeir Hellstrøm, CENTER-TBI Participants and Investigators, Cecilia Åkerlund, Krisztina Amrein, Lasse Andreassen, Audny Anke, Anna Antoni, Gérard Audibert, Philippe Azouvi, Maria Luisa Azzolini, Ronald Bartels, Pál Barzó, Romuald Beauvais, Ronny Beer, Bo-Michael Bellander, Antonio Belli, Habib Benali, Maurizio Berardino, Luigi Beretta, Morten Blaabjerg, Peter Bragge, Alexandra Brazinova, Vibeke Brinck, Joanne Brooker, Camilla Brorsson, Andras Buki, Monika Bullinger, Manuel Cabeleira, Alessio Caccioppola, Emiliana Calappi, Maria Rosa Calv, Peter Cameron, Guillermo Carbayo Lozano, Marco Carbonara, Simona Cavallo, Giorgio Chevallard, Arturo Chieregato, Giuseppe Citerio, Hans Clusmann, Mark Coburn, Jonathan Coles, Jamie D. Cooper, Marta Correia, Amra Čović, Nicola Curry, Endre Czeiter, Marek Czosnyka, Claire Dahyot-Fizelier, Paul Dark, Helen Dawes, Véronique DeKeyser, Vincent Degos, Francesco Della Corte, Hugo den Boogert, Bart Depreitere, Đula Đilvesi, Abhishek Dixit, Emma Donoghue, Jens Dreier, Guy-Loup Dulière, Ari Ercole, Patrick Esser, Erzsébet Ezer, Martin Fabricius, Valery L. Feigin, Kelly Foks, Shirin Frisvold, Alex Furmanov, Pablo Gagliardo, Damien Galanaud, Dashiell Gantner, Guoyi Gao, Pradeep George, Alexandre Ghuysen, Lelde Giga, Ben Glocker, Jagoš Golubovic, PedroA. Gomez, Johannes Gratz Benjamin Gravesteijn, Francesca Grossi, RussellL. Gruen, Deepak Gupta, JuanitaA. Haagsma, Iain Haitsma, Raimund Helbok, Lindsay Horton, Jilske Huijben, PeterJ. Hutchinson, Stefan Jankowski, Mike Jarrett, Ji-yao Jiang, Faye Johnson, Kelly Jones, Mladen Karan, AngelosG. Kolias, Erwin Kompanje, Daniel Kondziella, Evgenios Kornaropoulos, Lars-Owe Koskinen, Noémi Kovács, Ana Kowark, Alfonso Lagares, Linda Lanyon, Steven Laureys, Fiona Lecky, Didier Ledoux, Rolf Lefering, Valerie Legrand, Aurelie Lejeune, Leon Levi, Roger Lightfoot, Hester Lingsma, AndrewI. R. Maas, Ana M. Castaño-León, Marc Maegele, Marek Majdan, Alex Manara, Geoffrey Manley, Costanza Martino, Hugues Maréchal, Julia Mattern, Catherine McMahon, Béla Melegh, David Menon, Tomas Menovsky, Ana Mikolic, Benoit Misset, Visakh Muraleedharan, Lynnette Murray, Ancuta Negru, David Nelson, Virginia Newcombe, Daan Nieboer, József Nyirádi, Otesile Olubukola, Matej Oresic, Fabrizio Ortolano, Aarno Palotie, Paul M. Parizel, Jean-François Payen, Natascha Perera, Vincent Perlbarg, Paolo Persona, Wilco Peul, Anna Piippo-Karjalainen, Matti Pirinen, Dana Pisica, Horia Ples, Suzanne Polinder, Inigo Pomposo, Jussi P. Posti, Louis Puybasset, Andreea Radoi, Arminas Ragauskas, Rahul Raj, Malinka Rambadagalla, Isabel Retel Helmrich, Jonathan Rhodes, Sylvia Richardson, Sophie Richter, Samuli Ripatti, Saulius Rocka, Olav Roise, Jonathan Rosand, Jeffrey V. Rosenfeld, Christina Rosenlund, Guy Rosenthal, Rolf Rossaint, Sandra Rossi, Daniel Rueckert Martin Rusnák, Juan Sahuquillo, Oliver Sakowitz, Renan Sanchez-Porras, Janos Sandor, Nadine Schäfer, Silke Schmidt, Herbert Schoechl, Guus Schoonman, Rico Frederik Schou, Elisabeth Schwendenwein, Charlie Sewalt, Ranjit D. Singh, Peter Smielewski, Abayomi Sorinola, Emmanuel Stamatakis, Simon Stanworth, Robert Stevens, William Stewart, Ewout W. Steyerberg, Nino Stocchetti, Nina Sundström, Riikka Takala, Viktória Tamás, Tomas Tamosuitis, MarkSteven Taylor, Aurore Thibaut, Braden Te Ao, Olli Tenovuo, Matt Thomas, Dick Tibboel, Marjolein Timmers, Christos Tolias, Tony Trapani, CristinaMaria Tudora, Andreas Unterberg, Peter Vajkoczy, Shirley Vallance, Egils Valeinis, Zoltán Vámos, Mathieu van der Jagt, Gregory Van der Steen, Jeroen T. J. M. van Dijck, Inge A. M. van Erp, Thomas A. van Essen, Wim VanHecke, Caroline van Heugten, Ernest van Veen, Thijs Vande Vyvere, Roel P. J. van Wijk, Alessia Vargiolu, Emmanuel Vega, Kimberley Velt, Jan Verheyden, Paul M. Vespa, Anne Vik, Rimantas Vilcinis, Victor Volovici, Daphne Voormolen, Petar Vulekovic, KevinK. W. Wang, Daniel Whitehouse, Eveline Wiegers, Guy Williams, Lindsay Wilson, Stefan Winzeck, Stefan Wolf, Zhihui Yang, Peter Ylén, Frederick A. Zeiler, Veronika Zelinkova, Agate Ziverte, TommasoZoerle

**Affiliations:** 1 Department of Physical Medicine and Rehabilitation, Oslo University Hospital, Oslo, Norway; 2 Center for Habilitation and Rehabilitation Models and Services (CHARM), Institute of Health and Society, University of Oslo, Oslo, Norway; 3 Oslo Centre for Biostatistics and Epidemiology, Oslo University Hospital, Oslo, Norway; 4 Institute of Psychology, University of Innsbruck, Innsbruck, Austria; 5 Institute of Clinical Medicine, Faculty of Medicine, University of Oslo, Oslo, Norway; 6 Department of Neurosurgery, Division of Emergencies and Critical Care, Department of Research and Development, Oslo University Hospital, Oslo, Norway; 7 Department of Neuromedicine and Movement Science, NTNU - Norwegian University of Science and Technology, Trondheim, Norway; 8 Clinic of Rehabilitation, St. Olavs Hospital, Trondheim University Hospital, Trondheim, Norway; 9 NorHEAD - Norwegian Centre for Headache Research, Trondheim, Norway; 10 Department of Psychology, Norwegian University of Science and Technology, Trondheim, Norway; 11 TBI Network, Department of Psychology, Faculty of Health and Environmental Sciences, Auckland University of Technology, Auckland, New Zealand; 12 Neurological Rehabilitation Center Godeshöhe, Bonn, Germany; 13 Department of Traumatology & Department of Psychiatry, Psychotherapy, and Psychosomatics, Neuroscience Center Zurich, University of Zurich, University Hospital Zurich, Zürich, Switzerland; 14 Institute for Stroke and Dementia Research (ISD), University Hospital, Ludwig Maximilian University of Munich, Munich, Germany; 15 Department of Neurology, University of Groningen, University Medical Center Groningen, Groningen, The Netherlands; 16 Tanz Centre for Research in Neurodegenerative Diseases, University of Toronto, Toronto, ON Canada; 17 Canadian Concussion Centre, Krembil Brain Institute, Toronto, ON Canada; 18 Memory Clinic, Toronto Western Hospital, Toronto, ON Canada; 19 Department of Neurology, St. Olav University Hospital, Trondheim, Norway

**Keywords:** Post-traumatic headache, Brain trauma, Observational study, Secondary headache disorders

## Abstract

**Background:**

Headache is a prevalent and debilitating symptom following traumatic brain injury (TBI). Large-scale, prospective cohort studies are needed to establish long-term headache prevalence and associated factors after TBI. This study aimed to assess the frequency and severity of headache after TBI and determine whether sociodemographic factors, injury severity characteristics, and pre- and post-injury comorbidities predicted changes in headache frequency and severity during the first 12 months after injury.

**Methods:**

A large patient sample from the Collaborative European NeuroTrauma Effectiveness Research in Traumatic Brain Injury (CENTER-TBI) prospective observational cohort study was used. Patients were stratified based on their clinical care pathway: admitted to an emergency room (ER), a ward (ADM) or an intensive care unit (ICU) in the acute phase. Headache was assessed using a single item from the Rivermead Post-Concussion Symptoms Questionnaire measured at baseline, 3, 6 and 12 months after injury. Mixed-effect logistic regression analyses were applied to investigate changes in headache frequency and associated predictors.

**Results:**

A total of 2,291 patients responded to the headache item at baseline. At study enrolment, 59.3% of patients reported acute headache, with similar frequencies across all strata. Female patients and those aged up to 40 years reported a higher frequency of headache at baseline compared to males and older adults. The frequency of severe headache was highest in patients admitted to the ICU. The frequency of headache in the ER stratum decreased substantially from baseline to 3 months and remained from 3 to 6 months. Similar trajectory trends were observed in the ICU and ADM strata across 12 months. Younger age, more severe TBI, fatigue, neck pain and vision problems were among the predictors of more severe headache over time. More than 25% of patients experienced headache at 12 months after injury.

**Conclusions:**

Headache is a common symptom after TBI, especially in female and younger patients. It typically decreases in the first 3 months before stabilising. However, more than a quarter of patients still experienced headache at 12 months after injury. Translational research is needed to advance the clinical decision-making process and improve targeted medical treatment for headache.

**Trial registration:**

ClinicalTrials.gov NCT02210221.

**Supplementary Information:**

The online version contains supplementary material available at 10.1186/s10194-024-01751-0.

## Background

Posttraumatic headache (PTH) is a common symptom after traumatic brain injury (TBI) [1]. It is classified as a secondary headache, which develops within 7 days after trauma, regaining consciousness, or recovering the ability to sense and report pain [2]. The duration of PTH determines whether it is acute (less than 3 months) or persistent (more than 3 months) [2].

The reported prevalence of headache in the first year following TBI varies from 10 to 95%, depending on the severity of trauma, type of pain and other factors [3–6]. Applying a cutoff score of ≥ 2 (mild, moderate or severe problem) on the Rivermead Post-Concussion Symptoms Questionnaire (RPQ), 37.5% of patients with mild TBI reported headache at 6 months and almost 30% at 12 months [6]. Another prospective study estimated that 54% to 69% of patients with mild TBI experienced headache within the first year [3]. Several studies suggest that PTH is more frequent after mild TBI compared to moderate or severe TBI [7–9], while others show no association between injury severity and PTH [5, 10].

Although PTH tends to improve over time [6, 10–13], many patients still experience chronic headache regardless of TBI severity [14, 15, 9].

Conflicting evidence exists regarding the association between sex, age and PTH [3, 5, 16, 17]. In a prospective observational study of risk factors for PTH after mild TBI, female sex, younger age, presence of headache at the emergency department, and computed tomography (CT) abnormalities were significant risk factors for developing chronic PTH [16]. Studies on moderate to severe TBI cohorts [5] and all TBI severities [14] identified female sex as predicting persistent headache. Several studies have shown an association between PTH and mood disorders [10, 18–20]. Additionally, a migraine-like headache phenotype is common in patients affected by persistent PTH [21]. Moreover, a pre-injury history of migraine has been found to predict PTH [6, 19] and has been associated with its frequency, severity, and impact on activities of daily living following moderate and severe TBI [14].

Many headache types are associated with visual problems [22]. The visual system is likely to be directly affected along with the headache, or by the headache, or may even be part of its triggering mechanism [22]. Therefore, considering visual disturbances as a potential predictor of PTH seems reasonable. Concurrent damage to musculoskeletal structures, especially the head and neck, might result in tension-type headache. The presence of comorbid and concurrent neck pain with migraine has been shown to be a significant predictor of disability and physical limitations, associated with increased headache frequency, intensity and duration [23–26].

Considering the inconsistent findings in the literature, large-scale, prospective longitudinal studies on PTH across acute clinical care pathways and injury severities are necessary. These studies will provide critical knowledge for identifying factors that can cause and prolong PTH, which is crucial for improving intervention and targeted treatment for individuals experiencing PTH [27].

We used a large patient sample from the Collaborative European NeuroTrauma Effectiveness Research in Traumatic Brain Injury (CENTER-TBI) observational study. Patients were stratified according to clinical care pathway: admitted to an emergency room (ER), a ward (ADM) or an intensive care unit (ICU) in the acute phase [28]. Headache was assessed using a single item from the RPQ because this seems to provide a good estimate of the subjective experience of headache after TBI. In this study, we use the term “headache” rather than PTH as we did not use the International Classification of Headache Disorders (ICHD-3) headache classification [2]. The study aims were to:

1. Assess the frequency and severity of headache after TBI at baseline (i.e. time of study inclusion), as well as 3, 6 and 12 months after injury across age, sex, injury severity and clinical pathway.
2. Explore whether sociodemographic factors, injury severity characteristics, and pre- and post-injury comorbidities predicted changes in headache frequency and severity in the first 12 months after TBI.

We hypothesised that headache would be a significant burden for most patients after TBI, regardless of injury severity and time since injury.

## Methods

### Study design

Data were included from participants in the core study of the CENTER-TBI project, an observational, longitudinal cohort study of patients with all severities of TBI. The participants presented to 65 centres across Europe and Israel between December 19, 2014, and December 17, 2017. The included patients had a clinical diagnosis of TBI and an indication for CT scanning, presented to a study centre within 24h after injury, and provided informed consent according to local and national ethical and legal requirements. The exclusion criterion was having a severe pre-existing neurological disorder that could bias functional outcome assessments. Patients were differentiated prospectively into three strata according to the clinical care pathway: ER stratum (patients assessed in the ER and then discharged), ADM stratum (patients admitted to a hospital ward) and ICU stratum (patients who were primarily admitted to the ICU). The study protocol has been published elsewhere [28]. The trial was registered at ClinicalTrials.gov on August 6, 2014 (#NCT02210221). The STROBE checklist was used to report this study (see Additional file 7).

### Participants

The CENTER-TBI core study included 4,509 patients. This study analysed data from all patients in the ER, ADM and ICU strata who responded to the RPQ item measuring headache at least once, either at baseline (mean 2.5 days after admission to CENTER-TBI) or 3, 6 or 12 months after injury. In total, 2,291 patients (50.8% of patients in the core study) were included in this study (see Table 1 for baseline characteristics). Of these, 2,291 responded to the RPQ headache item at baseline, 2,162 at 3 months, 2,253 at 6 months and 1,450 at 12 months.

**Table 1:** Sociodemographic and clinical characteristics of the study population at baseline

**Characteristic**	Total (*N* = 2,291)	ER (*n* = 746)	ADM (*n* = 1,146)	ICU (*n* = 399)	*p*-value
**Sex, male %**	1,454 (63.5%)	417 (55.9%)	741 (64.7%)	296 (74.2%)	< 0.001
**Age, years**					< 0.001
Mean (SD)	48.8 (21.1)	47.3 (20.6)	50.8 (21.5)	45.8 (20.4)	
Median (IQR)	50 (30, 66)	46 (29, 64)	53 (33, 68)	46 (30, 62)	
**Age category, %**					< 0.001
0–18 years	166 (7.3%)	39 (5.2%)	86 (7.5%)	41 (10.4%)	
19–40 years	693 (30.2%)	269 (36.1%)	297 (25.9%)	127 (31.8%)	
41–65 years	849 (37.1%)	269 (36.1%)	427 (37.3%)	153 (38.3%)	
> 65 years	583 (25.4%)	169 (22.6%)	336 (29.3%)	78 (19.5%)	
**Education, years**					0.399
Mean (SD)	13.3 (4.3)	13.1 (4.0)	13.4 (4.4)	13.3 (4.8)	
Median (IQR)	13 (11, 16)	13 (10, 16)	13 (11, 16)	13 (11, 16)	
**Pre-injury ASA-PS, %**					< 0.001
Healthy	1,342 (58.9%)	428 (57.5%)	641 (56.3%)	273 (68.8%)	
Mild disease	720 (31.6%)	240 (32.2%)	385 (33.9%)	95 (23.9%)	
Severe disease	217 (9.5%)	77 (10.3%)	111 (9.8%)	29 (7.3%)	
**Pre-injury psychiatric problems, %**	(*n* = 2199) 279 (12.7%)	(*n* = 707) 107 (15.1%)	(*n* = 1107) 135 (12.2%)	(*n* = 385) 37 (9.6%)	0.025
**Previous TBI, %**	(*n* = 2,232) 259 (11.6%)	(*n* = 726) 107 (14.7%)	(*n* = 1118) 123 (11.0%)	(*n* = 388) 29 (7.5%)	0.001
**Pre-injury migraine treatment, %**	(*n* = 1,231) 134 (10.9%)	(*n* = 375) 45 (12.0%)	(*n* = 612) 68 (11.1%)	(*n* = 244) 21 (8.6%)	0.403
**Cause of injury, %**					< 0.001
Incidental fall	1,118 (50.9%)	363 (50.1%)	576 (52.3%)	179 (48.0%)	
Traffic accident	768 (34.9%)	241 (33.3%)	373 (33.9%)	154 (41.2%)	
Others	312 (14.2%)	120 (16.6%)	152 (13.8%)	40 (10.8%)	
**GCS category, %**					< 0.001
GCS 13–15	2,099 (93.3%)	735 (99.6%)	1,101 (98.0%)	263 (67.6%)	
GCS 9–12	64 (2.8%)	2 (0.3%)	18 (1.6%)	44 (11.3%)	
GCS 3–8	87 (3.9%)	1 (0.1%)	4 (0.4%)	82 (21.1%)	
**Brain Injury AIS (≥ 3), %**	(*n* = 2283) 1,204 (52.7%)	(*n* = 746) 62 (8.3%)	(*n* = 1140) 797 (69.9%)	(*n* = 397) 345 (86.9%)	< 0.001
**ISS, median (IQR)**	9 (2, 16)	4 (2, 8)	10 (9, 16)	24 (16, 33)	< 0.001
**ISS face injury, yes, %**	562 (24.5%)	161 (21.6%)	284 (24.8%)	117 (29.3)	0.014
**CT head: presence of intracranial injury, %**	(*n* = 2182) 694 (31.8%)	(*n* = 701) 67 (9.6%)	(*n* = 1101) 375 (34.1%)	(*n* = 380) 252 (66.3%)	< 0.001
**RPQ, feeling depressed (≥ 2), %**	(*n* = 2285) 425 (18.6%)	(*n* = 745) 130 (17.4%)	(*n* = 1142) 202 (17.7%)	(*n* = 398) 93 (23.4%)	0.027
**RPQ, sleep problems (≥ 2), %**	(*n* = 2258) 620 (27.5%)	(*n* = 727) 127 (17.5%)	(*n* = 1133) 344 (30.4%)	(*n* = 398) 149 (37.4%)	< 0.001
**RPQ, fatigue (> 2), %**	(*n* = 2286) 1,071 (46.9%)	(*n* = 745) 291 (39.1%)	(*n* = 1142) 550 (48.2%)	(*n* = 399) 230 (57.6%)	< 0.001
Vision problems*, %	(*n* = 2110) 487 (23.1%)	(*n* = 465) 93 (20.0%)	(*n* = 816) 169 (20.7%)	(*n* = 829) 225 (27.1%)	0.002
Neck pain*, %	(*n* = 2110) 684 (32.4%)	(*n* = 466) 142 (30.5%)	(*n* = 815) 273 (33.5%)	(*n* = 829) 269 (32.4%)	0.415
Mobility problems*, %	(*n* = 2113) 577 (27.3%)	(*n* = 466) 72 (15.5%)	(*n* = 816) 198 (24.4%)	(*n* = 831) 307 (36.9%)	< 0.001

*Values not available at baseline and represent 3-month follow-up

*Abbreviations: ER* Emergency room stratum, *ADM* Admission stratum (hospital ward), *ICU* Intensive care unit stratum*, ASA-PS* American Society of Anesthesiologists Physical Status Classification System score, *GCS* Glasgow Coma Scale, *AIS* Abbreviated Injury Scale, *ISS* Injury Severity Score, *CT* Computed tomography, *RPQ* The Rivermead Post-Concussion Symptoms Questionnaire

### Ethical approval

This study was approved by the CENTER-TBI management committee. The CENTER-TBI study (EC grant 602,150) was conducted following all relevant European Union laws and all relevant laws of the country in which the recruiting sites were located. The informed consent of the patients, their legal representative or next of kin was obtained according to the local legislation for all patients recruited in the core dataset of CENTER-TBI and documented in the electronic case report form. For the full list of sites and ethical committees, see the official CENTER-TBI website (https://www.center-tbi.eu/project/ethical-approval).

### Measurements

#### Independent variables

Sociodemographics: age, sex and education were collected at study admission. Injury severity characteristics were also recorded: patient stratum; injury mechanism; *Glasgow Coma Scale* (GCS) score within the first 24h after injury [29]; presence of intracranial injuries on first CT head scan; Brain Injury Score using the *Abbreviated Injury Scale* (Brain injury AIS; score ≥ 3 considered severe injury) [30], and *Injury Severity Score* (ISS), where a score of > 15 was considered major overall trauma [31], in addition to injuries to the face (ISS Face).

Pre- and post-injury comorbidities: Pre-injury somatic comorbidities were classified according to the *American Society of Anesthesiologists Physical Status Classification System* score (ASA-PS) [32]. Premorbid psychiatric problems comprised anxiety, depression, sleep disorders, schizophrenia, drug abuse or other psychiatric problems as reported by patients retrospectively at follow-up. Information on pre-injury migraine treatment or family history of migraine was also collected retrospectively using questionnaires at the study enrolment.

Three additional items from the RPQ were used to assess fatigue, sleep disturbance and feeling depressed at baseline, applied as determinants of post-injury comorbidities potentially relevant to headache. A cutoff score of ≥ 2 (mild, moderate or severe problem) was used. Neck pain, vision problems and problems with mobility were reported retrospectively 3 months after injury on a questionnaire specifically designed for data collection in the CENTER-TBI study. All outcome instruments used in the CENTER-TBI study can be found on the official website (https://www.center-tbi.eu/project/validated-translations-outcome-instruments).

### Outcome variable

Headache was measured at baseline and 3, 6 and 12 months of follow-up using a single item from the RPQ, measured on a 5-point scale (0 = not a problem, 1 = no more of a problem than before, 2 = mild problem, 3 = moderate problem, 4 = severe problem) [33]. Cutoff values of ≥ 2, corresponding to a mild, moderate or severe problem, in addition to ≥ 3, corresponding to moderate or severe symptoms, were applied. Responses rated as 1 were recoded as 0 according to the originally proposed scoring of the RPQ. The first assessment of headache was performed at study admission, a mean of 2.5 (standard deviation [SD] 12) days after the onset. Both adults (age ≥ 16 years) and children or their parents (age < 16 years) were asked to rate the severity of headache compared to their pre-injury status during the last 7 days. The RPQ is a reliable measure of symptoms commonly experienced after TBI [33, 34], and a study assessing the validity of the RPQ showed that the questionnaire was unbiased for an age range of 6–96 years [35], and parent ratings of fatigue in children with TBI have been applied in previous research [36]. If the participants reported headache at the follow-ups, it was considered persistent headache. The data were collected in face-to-face interviews or by postal or electronic questionnaires.

### Statistical analyses

The CENTER-TBI dataset version 2.0 (from May 2019) was analysed in this study. For descriptive statistics, means with SDs, medians with interquartile ranges (IQRs), or percentages are presented. Differences in demographic and injury-related data between patient strata (ER, ADM and ICU) were tested using a one-way analysis of variance for normally distributed continuous variables or Kruskal–Wallis test for continuous variables with skewed distribution. A chi-square test for contingency tables was performed to detect group differences in categorical variables.

To investigate changes in headache frequency between the patient strata over the entire follow-up period and account for repeated measures by patient, mixed-effect logistic regression was performed using headache (dichotomised at the value ≥ 2) as the outcome variable. Since only patients in the ADM and ICU strata were followed up to 12 months after injury, two separate models were performed: one comparing all patient strata (ER, ADM and ICU) up to 6 months post injury (baseline and 3 and 6 months) and a second model only comparing ADM and ICU up to 12 months post injury (baseline and 3, 6 and 12 months). Time, patient stratum and time-by-patient stratum interaction were introduced as fixed effects in all models. Based on the mixed-effects logistic regression, we estimated risk differences with 95% confidence intervals (CIs) from baseline to 6 months using the delta method. For comparison of the effects of different cutoffs, the analysis was also performed using headache dichotomised at the values of ≥ 3 as the outcome variable.

Mixed-effect logistic regression analyses were performed to investigate whether changes in the proportion reporting headache (dichotomised at the value of ≥ 2 or ≥ 3) during the follow-up period could be predicted by age, sex, patient stratum, education, pre-injury ASA-PS and psychiatric comorbidities, GCS score, intracranial injury on CT, Brain Injury AIS, ISS (ISS Face was omitted due to high correlation with ISS), and the RPQ items “feeling depressed”, “fatigue” and “sleep disturbance” (dichotomised at the value of ≥ 2) measured at baseline. Additionally, visual problems, neck pain and mobility problems measured at 3 months after injury were assessed as predictors. As described, two different models were performed with different follow-up times. Time and all predictor variables were treated as fixed effects in the models. Interaction effects between time and fixed factors were verified by introducing product terms. All models included a random intercept.

Missing outcome data were handled by mixed-effects logistic regression models (i.e. no imputation was required). Missing predictor data were handled by multiple imputations, generating 10 imputed datasets, applying the multiple imputation by chained equations procedure in Stata [37]. The mixed-effect logistic regression models were repeated in the 10 imputed datasets, and results were pooled using Rubin’s rule.

All statistical analyses were performed using IBM SPSS Statistics for Windows version 29 (Armonk, NY, USA: IBM Corp.) and Stata 17 (Stata Corp LLC, College Station, TX, USA). A p*-*value of < 0.05 was considered statistically significant in all analyses.

## Results

A total of 2,291 patients responded to the headache item at baseline. Table 1 shows the demographic and injury characteristics by patient strata; 746 patients were included in the ER stratum, 1,146 in ADM and 399 in ICU. The median age of the total sample was 50 (IQR 30, 66) years, and 63.5% of the participants were male. The median education was 13 (IQR 11, 16) years. Sociodemographic and injury severity characteristics differed significantly between patient strata. According to GCS score, 93.3% of the total sample had sustained mild, 2.8% moderate and 3.9% severe TBI. Regarding the proportion who reported receiving treatment for pre-injury migraine, no statistically significant differences existed between the ER (12.0%), ADM (11.1%) and ICU (8.6%) strata.

Table 2 shows the proportion of patients in each stratum who reported headache at each cutoff level at baseline. Applying a cutoff score of ≥ 2 (mild problem) revealed that a total of 59.3% reported headache at baseline. When using a more conservative cutoff score of ≥ 3 (indicating a moderate or severe problem), the frequency was 29.3%. The median RPQ headache score was similar across all patient strata (2, IQR 0–3, *p* = 0.121), with no significant between-strata differences in the frequency who reported headache when applying cutoffs of ≥ 2 and ≥ 3. However, additional analysis of patients who reported severe headache only (cutoff 4) showed significant differences between the strata (*p* = 0.030), with the highest frequency in patients admitted to the ICU (11.5% vs 7% in patients admitted to the ER). The proportion of patients in each stratum who reported headache at 3, 6 and 12 months of follow-up is presented in Additional File 1.

**Table 2:** Headache frequency at baseline by patient stratum and RPQ headache severity score

RPQ headache score at baseline	Total (*n* = 2,291)	ER (*n* = 746)	ADM (*n* = 1,146)	ICU (*n* = 399)	*p*-value
Median (IQR)	2 (0, 3)	2 (0, 3)	2 (0, 3)	2 (0, 3)	0.121
RPQ headache score ≥ 2	1,359 (59.3%)	434 (58.2%)	697 (60.8%)	228 (57.1%)	0.324
RPQ headache score ≥ 3	671 (29.3%)	206 (27.6%)	360 (31.4%)	105 (26.3%)	0.074
RPQ headache score 4	204 (8.9%)	52 (7.0%)	106 (9.2%)	46 (11.5%)	0.030

The proportion who reported RPQ headache score < 2 is not presented in the table

*Abbreviations*: *RPQ* The Rivermead Post-Concussion Symptoms Questionnaire, *ER* Emergency room stratum, *ADM* Admission stratum (hospital ward), *ICU* Intensive care unit stratum, *IQR* interquartile range

**Fig. 1:**
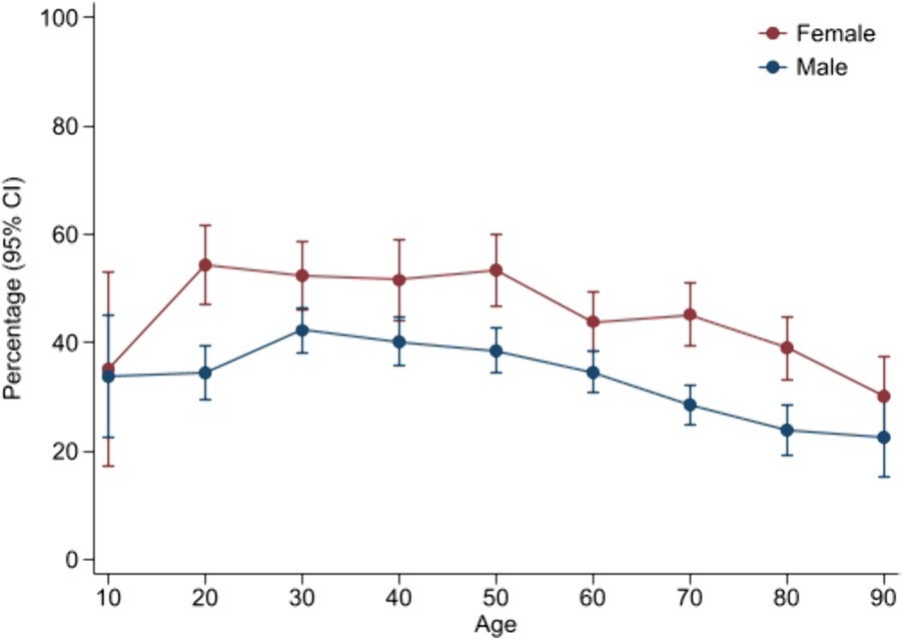
Frequency of patients with headache (RPQ cutoff ≥ 2) at baseline by 10-year age interval and sex

Figure 1 shows the frequency of reporting headache (cutoff ≥ 2) at baseline by sex and 10-year age interval. The frequency was highest in female patients across all age groups, with a total of 66.9% of females and 55.0% of males reporting headache. The highest frequency of moderate or severe headache (≥ 3) was found in women aged 20–29 years (17.6%) and men aged 20–29 years (21.2%). The lowest was in both female and male patients aged 0–9 years (0% and 1.1%, respectively) and 90–99 years (1.0% and 0%, respectively). Applying cutoff ≥ 3, revealed that 37.3% of female patients and 24.7% of male patients reported headache.

### Change in headache frequency across 6 and 12 months follow-up

**Fig. 2:**
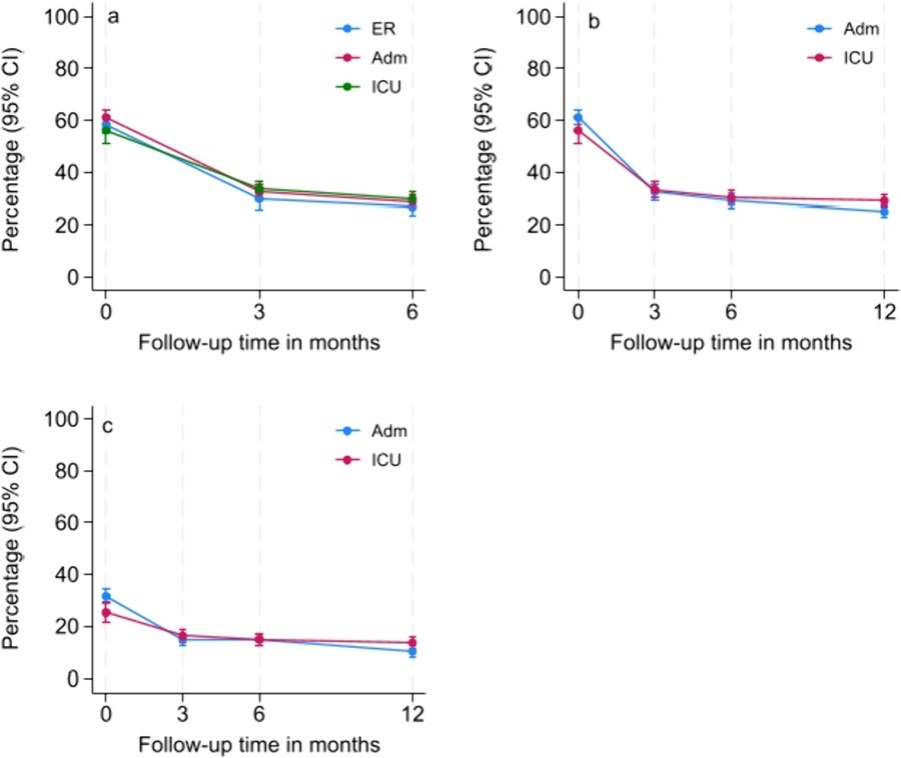
**a** Estimated proportions of patients with headache (cutoff ≥ 2) by ER, ADM and ICU strata up to 6 months, **b** Estimated proportions of patients with headache (cutoff ≥ 2) by ADM and ICU strata up to 12 months, **c** Estimated proportions of patients with headache (cutoff ≥ 3) by ADM and ICU strata up to 12 months

The estimated proportion of patients reporting RPQ headache scores of ≥ 2 by patient strata up to 6 months (all strata), in addition to scores of ≥ 2 and ≥ 3 up to 12 months (ADM and ICU strata), are reported in Fig. 2. Statistically significant decreases occurred in the proportions of individuals reporting headache from baseline to 3, 6 and 12 months within all strata. The same tendency was observed when assessing change in headache frequency using GCS score to classify mild, moderate and severe TBI instead of patient strata, and results showed no statistically significant between-group change in estimated proportion of patients with RPQ cutoff ≥ 2 from baseline to 12 months follow-up (see Additional file 2). No statistically significant change occurred in the estimated proportion reporting headache (cutoffs of ≥ 2 or ≥ 3) in the ER, ADM and ICU strata across the first 6 months after injury. However, a statistically significant change occurred between the ADM and ICU strata from baseline to 12 months follow-up using a cutoff of ≥ 2 (mean change 0.08, 95% CI 0.02 to 0.15, p = 0.006). Applying a RPQ headache score of ≥ 3 additionally showed a statistically significant change between the ADM and ICU strata from baseline to 3 months (mean change 0.07, 95% CI 0.02 to 0.12, *p* = 0.005).

### Predictors of change in headache severity

We used four models to assess whether change in the proportion who reported RPQ headache scores of ≥ 2 and ≥ 3 could be predicted by demographic variables, injury severity indicators and comorbid conditions up to 6 (model 1 and 2, all strata) and 12 (model 3 and 4, ICU and ADM strata) months after injury. All statistically significant and non-significant fixed effects from the full model and their coefficients, *p*-values and 95% CIs are presented in Tables 3 and 4.

**Table 3:** Predictors of change in headache in the first 6 months after injury (all strata)

	Model 1 (RPQ cutoff ≥ 2)			Model 2 (RPQ cutoff ≥ 3)		
	Coef	95% CI	*p*-value	Coef	95% CI	*p*-value
**Intercept**	1.13***	0.61 to 1.65	< 0.001	-0.83**	-1.35 to -0.31	0.002
**Time**	-0.38***	-0.51 to -0.25	< 0.001	-0.21**	-0.35 to -0.07	0.002
**Patient stratum**						
ER	Ref			Ref		
ADM	-0.16	-0.49 to 0.16	0.331	-0.12	-0.47 to 0.22	0.478
ICU	-0.18	-0.69 to 0.31	0.464	-0.24	-0.77 to 0.28	0.358
**Age,** y	-0.01***	-0.02 to -0.01	< 0.001	-0.01***	-0.02 to -0.01	< 0.001
**Sex** (f = 0, m = 1)	-0.55***	-0.80 to -0.29	< 0.001	-0.52***	-0.77 to -0.26	< 0.001
**Preinjury ASA-PS**						
Healthy	Ref			Ref		
Mild disease	0.18	-0.10 to 0.47	0.219	0.14	-0.14 to 0.44	0.325
Severe disease	-0.45*	-0.90 to 0.003	0.052	-0.12	-0.60 to 0.36	0.621
**Pre-injury psychiatric**	-0.05	-0.42 to 0.30	0.750	0.49**	0.13 to 0.85	0.007
**Pre-injury migraine**	0.27	-0.15 to 0.70	0.202	0.35	-0.17 to 0.88	0.180
**GCS (3–15)**	-0.16	-0.43 to 0.11	0.249	-0.04	-0.34 to 0.25	0.772
**CT head intracranial injury**	0.07	-0.22 to 0.37	0.614	0.17	-0.11 to 0.46	0.243
**Brain Injury AIS (≥ 3)**	1.01***	0.67 to 1.36	< 0.001	0.95***	0.59 to 1.30	< 0.001
**ISS**	-0.04***	-0.06 to -0.03	< 0.001	-0.04***	-0.06 to -0.02	< 0.001
**Feeling depressed** at baseline **(≥ 3)**	0.63***	0.30 to 0.96	< 0.001	0.53*	0.07 to 0.99	0.023
** Sleep disturbance** at baseline **(≥ 3)**	0.31*	0.01 to 0.61	0.037	0.84***	0.48 to 1.20	< 0.001
**Fatigue** at baseline **(≥ 3)**	0.73***	0.47 to 0.99	< 0.001	0.99***	0.68 to 1.29	< 0.001
**Neck pain**	0.89***	0.62 to 1.17	< 0.001	0.79***	0.50 to 1.09	< 0.001
** Vision problems**	0.37*	0.04 to 0.69	0.026	0.48**	0.15 to 0.82	0.005
**Mobility problems**	-0.18	-0.53 to 0.17	0.308	-0.14	-0.48 to 0.19	0.403
**Time x Predictors**						
**Time x ICU**	0.08	-0.03 to 0.20	0.181	0.10	-0.03 to 0.24	0.150
**Time x Age**	-0.001	-0.002 to 0.0005	0.178	-0.01	-0.003 to 0.0007	0.230
**Time x Sex**	-0.02	-0.08 to 0.03	0.486	0.01	-0.05 to 0.07	0.837
**Time x Pre-injury ASA-PS**						
Time x Mild disease	0.03	-0.03 to 0.10	0.354	0.01	-0.06 to 0.09	0.709
Time x Severe disease	0.21***	0.10 to 0.33	< 0.001	0.06	-0.06 to 0.19	0.313
**Time x Pre-injury psychiatric**	0.08*	-0.0002 to 0.17	0.051	-0.02	-0.11 to 0.07	0.662
**Time x Pre-injury migraine**	0.02	-0.07 to 0.11	0.663	0.01	-0.11 to 0.12	0.946
**Time x GCS**	0.0003	-0.06 to 0.06	0.991	-0.03	-0.10 to 0.02	0.265
**Time x CT head intracranial injury**	0.03	-0.04 to 0.10	0.407	0.01	-0.06 to 0.09	0.737
**Time x Brain Injury AIS**	-0.10*	-0.19 to -0.01	0.019	-0.10*	-0.20 to -0.01	0.043
**Time x ISS**	0.005**	0.001 to 0.009	0.003	0.01*	0.0007 to 0.008	0.019
**Time x Feeling depressed**	-0.04	-0.12 to 0.03	0.305	0.02	-0.09 to 0.15	0.659
**Time x Sleep disturbance**	0.02	-0.05 to 0.10	0.529	-0.9	-0.19 to 0.008	0.071
**Time x Fatigue**	-0.12**	-0.20 to -0.04	0.002	-0.16***	-0.25 to -0.08	< 0.001
**Time x Neck pain**	0.12**	0.05 to 0.19	0.001	0.15***	0.08 to 0.23	< 0.001
**Time x Vision**	0.09*	0.01 to 0.16	0.016	0.05	-0.03 to 0.13	0.216
**Time x Mobility**	0.07	-0.004 to 0.15	0.065	0.11**	0.02 to 0.19	0.009

*6-*month follow-up all strata. Model 1: Headache cutoff ≥ 2, Model 2: Headache ≥ 3

*Abbreviations: ER* Emergency room stratum, *ADM* Admission stratum (hospital ward), *ICU* Intensive care unit stratum, ASA-PS American Society of Anesthesiologists Physical Status Classification System score, *GCS* Glasgow Coma Scale, *CT* Computed tomography, *AIS* Abbreviated Injury Scale, *ISS* Injury Severity Score

^*^ = *p* < 0.05; ** = *p* < 0.01; *** = *p* < 0.001

**Table 4:** Predictors of change in headache in the first 12 months post injury (ADM and ICU)

	Model 3 (RPQ cutoff ≥ 2)			Model 4 (RPQ cutoff ≥ 3)		
	Coef	95% CI	*p*-value	Coef	95% CI	*p*-value
**Intercept**	0.50	-0.08 to 1.08	0.092	-1.40***	-1.99 to -0.80	< 0.001
**Time**	-0.14***	-0.23 to -0.06	< 0.001	-0.08	-0.17 to 0.007	0.070
**Patient stratum**						
ADM	Ref			Ref		
ICU	-0.10	-0.47 to 0.25	0.560	-0.11	-0.50 to 0.27	0.574
**Age,** y	-0.01***	-0.02 to -0.009	< 0.001	-0.01**	-0.01 to -0.004	0.002
**Sex** (f = 0, m = 1)	-0.49**	-0.77 to -0.21	0.001	-0.39**	-0.67 to -0.10	0.008
**Preinjury ASA-PS**						
Healthy	Ref			Ref		
Mild disease	0.19	-0.12 to 0.51	0.236	0.03	-0.30 to 0.37	0.838
Severe disease	-0.12	-0.63 to 0.39	0.640	-0.22	-0.78 to 0.33	0.433
**Pre-injury psychiatric**	-0.21	-0.62 to 0.19	0.299	0.42*	0.01 to 0.83	0.043
**Pre-injury migraine**	0.31	-0.22 to 0.85	0.193	0.33	-0.24 to 0.91	0.257
**GCS (3–15)**	-0.13	-0.36 to 0.10	0.282	-0.10	-0.36 to 0.15	0.416
**CT head intracranial injury**	0.02	-0.26 to 0.31	0.868	0.13	-0.16 to 0.43	0.386
**Brain Injury AIS (≥ 3)**	0.93***	0.58 to 1.28	< 0.001	0.90***	0.53 to 1.28	< 0.001
**ISS**	-0.04***	-0.06 to -0.03	< 0.001	-0.04***	-0.05 to -0.02	< 0.001
**Feeling depressed** at baseline	0.57**	0.19 to 0.96	0.003	0.78**	0.22 to 1.35	0.006
**Sleep disturbance** at baseline	0.28	-0.09 to 0.66	0.133	0.83***	0.43 to 1.24	< 0.001
**Fatigue** at baseline	0.59***	0.31 to 0.87	< 0.001	0.76***	0.40 to 1.12	< 0.001
**Neck pain**	1.03***	0.74 to 1.32	< 0.001	0.85***	0.53 to 1.18	< 0.001
**Vision problems**	0.45**	0.12 to 0.77	0.006	0.44*	0.10 to 0.78	0.011
**Mobility problems**	-0.15	-0.49 to 0.18	0.363	0.03	-0.30 to 0.37	0.850
**Time x Predictors**						
**Time x ICU**	0.06*	0.01 to 0.11	0.013	0.05	-0.001 to 0.11	0.055
**Time x Age**	-0.001**	-0.002 to -0.0006	0.002	-0.002**	-0.003 to -0.0008	0.001
**Time x Sex**	-0.02	-0.06 to 0.01	0.240	-0.03	-0.08 to 0.006	0.092
**Time x Pre-injury ASA-PS**						
Time x Mild disease	0.01	-0.03 to 0.05	0.628	0.01	-0.03 to 0.07	0.507
Time x Severe disease	0.06	0.007 to 0.13	0.081	0.01	-0.07 to 0.10	0.747
**Time x Preinjury psychiatric**	0.04	-0.01 to 0.09	0.157	0.001	-0.06 to 0.06	0.963
**Time x Migraine**	-0.02	-0.09 to 0.04	0.488	-0.03	-0.11 to 0.04	0.433
**Time x GCS**	-0.01	0.04 to 0.01	0.278	-0.01	-0.05 to 0.01	0.344
**Time x CT head intracranial injury**	0.02	-0.02 to 0.06	0.310	0.02	-0.02 to 0.07	0.257
**Time x Brain Injury AIS**	-0.04	0.09 to 0.007	0.090	-0.04	-0.11 to 0.01	0.137
**Time x ISS**	0.003**	0.001 to 0.005	0.001	0.003**	0.001 to 0.005	0.001
**Time x Feeling depressed**	-0.03	-0.08 to 0.02	0.233	-0.05	-0.15 to 0.03	0.248
**Time x Sleep disturbance**	0.03	-0.01 to 0.07	0.183	-0.04	-0.11 to 0.02	0.192
**Time x Fatigue**	-0.03	-0.08 to 0.01	0.121	-0.07**	-0.13 to -0.02	0.004
**Time x Neck pain**	0.04*	0.005 to 0.09	0.025	0.00***	0.04 to 0.13	< 0.001
**Time x Vision**	0.01	-0.03 to 0.05	0.615	0.01	-0.04 to 0.06	0.703
**Time x Mobility**	0.06**	0.02 to 0.11	0.002	0.04	-0.004 to 0.09	0.076

*12-month follow-up ADM and ICU strata Model 3: Headache cutoff* ≥ *2, Model 4: Headache* ≥ *3*

*Abbreviations: ADM* Admission stratum (hospital ward), *ICU* Intensive care unit stratum, *ASA-PS* American Society of Anaesthesiologists Physical Status Classification System score*, GCS* Glasgow Coma Scale*, CT* Computed tomography*, AIS* Abbreviated Injury Scale, *ISS* Injury Severity Score

^*^= *p* < 0.05; ** = *p* < 0.01; *** = *p* < *0.001*

In model 1 (cutoff ≥ 2), time, age, sex, pre-injury ASA, Brain Injury AIS, ISS, depressive symptoms, sleep disturbance, fatigue, neck pain and vision problems yielded significant effects on the probability of headache frequency over the first 6 months after injury. Younger patients, female patients, and those with severe pre-morbid somatic disease, more severe head injury (Brain Injury AIS) and less severe overall injury (ISS), post-injury comorbidity (depressive symptoms and sleep disturbance), fatigue, presence of neck pain and vision problems, and shorter time since injury were more likely to have headache. Except for pre-injury ASA, the same predictors remained statistically significant in model 2 (cutoff ≥ 3). Additionally, patients with pre-injury psychiatric conditions had a statistically significantly higher probability of headache when applying a cutoff of ≤ 3.

In model 3 (cutoff ≥ 2), time, age, sex, Brain Injury AIS, ISS, depressive symptoms, fatigue, neck pain and vision problems were significant predictors of headache probability across the first 12 months after injury. A shorter time since injury, younger age, female sex, more severe head injury, less severe overall injury (ISS), more depressive symptoms and fatigue, presence of neck pain and vision problems predicted a higher probability of headache. When applying a cutoff of ≥ 3 (model 4), age, sex, Brain Injury AIS, ISS, depressive symptoms, fatigue, neck pain and vision problems remained statistically significant predictors. Additionally, a statistically significantly higher probability of headache existed in patients with sleep problems and premorbid psychiatric conditions.

### Significant interaction effects between time and predictors

**Fig. 3:**
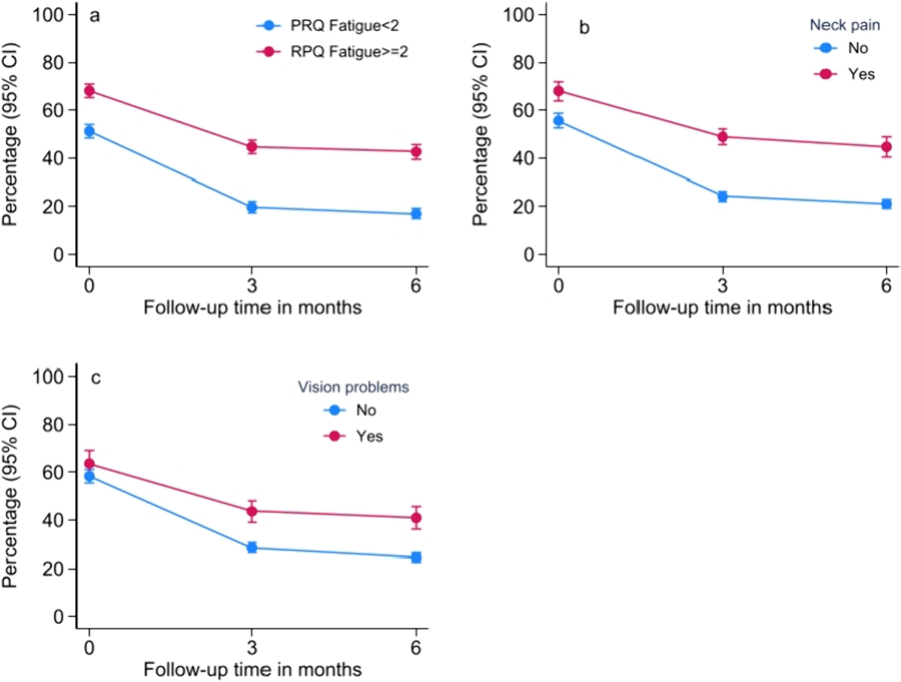
**a** Main effect and time interaction of fatigue on headache changes, **b** main effect and time interaction of neck pain on headache changes, **c** main effect and time interaction of vision problems on headache changes

In model 1 (all strata, cutoff ≥ 2), statistically significant interaction effects existed between time and pre-injury ASA, pre-injury psychiatric disease, Brain Injury AIS, ISS, fatigue (Fig. 3a), neck pain (Fig. 3b) and vision problems (Fig. 3c). The significant interaction effect between time and pre-morbid somatic disease suggested that patients with severe pre-morbid somatic disease tended to initially report lower headache, with a decrease from baseline to 3 months, and thereafter increased from 3 to 6 months of follow-up. Patients with no or mild pre-injury somatic disease initially reported more headache and thereafter a steady decrease from baseline to 3 and 6 months. Patients with less severe overall injury (ISS) initially tended to report more headache but showed a steeper decline in headache from 3 to 6 months. Additionally, patients with pre-injury psychiatric problems, more severe head injury, fatigue, neck pain and vision problems had a higher probability of headache over the first 6 months after injury. In model 3 (all strata, cutoff ≥ 3), the same variables remained statistically significant, except for vision problems and pre-injury ASA, which were no longer significant. Additionally, patients without mobility problems initially reported more headache but thereafter a steeper decline and lower headache than patients with mobility problems at 3 and 6 months.

**Fig. 4:**
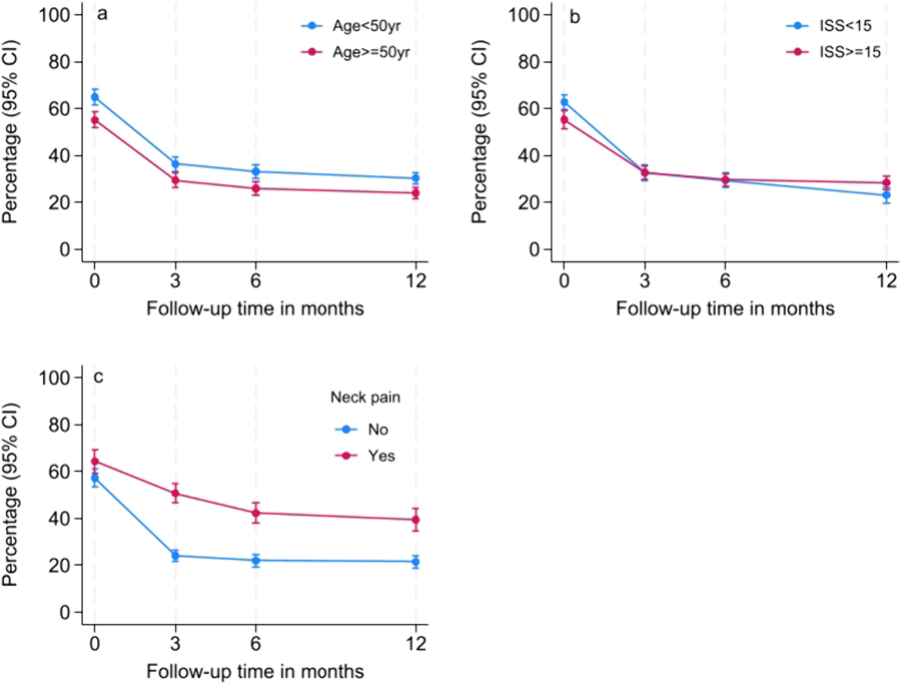
**a** Main effect and time interaction of age on headache changes, **b** main effect and time interaction of ISS on headache changes, **c** main effect and time interaction of neck pain on headache changes

In model 3 (ADM and ICU strata, cutoff > 2), we found statistically significant interaction effects between time and ICU stratum, age, ISS, neck pain and mobility. Younger age (Fig. 4a), more severe fatigue and neck problems (Fig. 4c) were associated with a higher probability of headache across the first 12 months after injury. Patients with mobility problems tended to report the same degree of headache as those without mobility problems at baseline, but the trajectory diverged somewhat thereafter, showing that patients with mobility problems reported more headache over time. Moreover, patients with less severe overall injury (ISS) tended to initially report more headache but a steeper decline in headache than patients with more severe overall injury from 6 to 12 months, as well as lower headache than patients with more severe injury at 12 months of follow-up (Fig. 4b). In model 4 (ADM and ICU strata, cutoff 3), the same variables remained statistically significant, except for ICU stratum and mobility problems, which were no longer significant. Moreover, patients with more severe fatigue consistently reported more headache and slower improvement than patients without fatigue. All statistically significant main effects and time interactions of the variables not presented in the main text are presented in Additional files 3, 4, 5 and 6.

## Discussion

Using data from the large-scale, observational CENTER-TBI study, we assessed the frequency of acute and persistent headache following TBI, changes in headache frequency across clinical care pathways, severity of injury and predictors of headache in the first year after TBI.

### Headache frequency and severity across the first 12 months after injury

As hypothesised, we found a high frequency of headache throughout the whole sample included in this study: 59.3% of patients reported acute headache (RPQ cutoff ≥ 2) at study enrolment, with similar frequencies across all strata. The proportion of patients in the ER stratum who reported headache (58.2%) aligns with previous studies on mild TBI, including TRACK-TBI [27], which reported a prevalence of 60.4% at 2 weeks after the injury, as well as the Dutch UPFRONT study (51.0%) [38].

As expected, the proportion of headache in the ER stratum decreased substantially from baseline to 3 months and remained stable from 3 to 6 months. Similar trajectory trends were observed in the ICU and ADM strata across 12 months. However, more than 25% of patients experienced headache at 12 months after injury. These findings suggest that interventions to improve the experience of headache are needed if symptoms have not resolved by 3 months [27]. Current treatment approaches to headache after TBI are guided by phenotypic expression and the use of headache diaries to assess treatment effectiveness. Factors that contribute to the chronicity of PTH include the characteristics of head trauma, previous history of headache from head trauma, presence of posttraumatic stress disorder, other mental health conditions and other comorbidities. Therefore, the management of PTH should be multidisciplinary. However, a lack of understanding regarding the relevance of these factors and to what extent they influence the treatment response has impeded the development of specific treatment approaches [39].

Female and younger (≤ 40 years) patients reported a higher frequency of headache at baseline compared to males and older adults. The frequency of severe headache (RPQ cutoff 4) was highest in patients admitted to the ICU. Our results suggest that more severe TBI may increase the risk of headache, probably due to neuro-morphological brain changes. Hoffman et al. also reported that TBI severity (based on the duration of post-traumatic amnesia) was a risk factor for the development of persistent headache [5]. However, this contrasts with previous research that reported an increased risk of headache in those with mild TBI [9, 40]. Several reasons could explain this. Patients with more severe injuries can be reasonably assumed to have a higher risk of experiencing headache, both from peripheral sources such as the dura mater and bone, which are innervated by pain receptors called nociceptors [4], and from central sources, such as lesions in the central somatosensory nervous system [41]. Additionally, PTH is widely prevalent after mild TBI, which may be attributed to differences in its diagnosis among studies, the length of time between the injury and evaluation, and the specific population being tested (e.g. civilians or veterans) [4]. The patients in this study were mainly recruited from trauma referral centres and, therefore, were on the more severe end of the mild TBI spectrum.

Findings regarding the association between headache and demographic factors are inconsistent in the literature [27]. In this study, younger age and female sex were associated with more severe headache, aligning with results from other large-scale studies [27]. The association between younger age and higher levels of headache may reflect the TBI severity in this population (9.4% of patients aged ≤ 40 years had moderate or severe TBI, in contrast to 5.2% of patients aged > 40 years). Previous studies have also found female sex to be associated with headache after TBI [5, 14, 16, 17].

### Predictors of change in headache frequency and severity in the first 12 months after injury

The present results also support a relationship between headache and more severe TBI. This was indicated by the significant Brain Injury AIS score, which was associated with the headache severity level. This aligns with a study that found a dose–response relationship between TBI severity and headache outcomes [42]. However, we also found a significant association between less severe overall injury (ISS) and more severe headache. Looking further into this association revealed that although patients with less severe overall injuries initially reported more headache, they subsequently had a steeper decline in symptoms, and at 12 months, they reported less headache than patients with more severe overall injuries. A possible explanation for this may be that patients with more severe overall injuries are provided with more analgetics in the acute stage, which may prevent pain and headache sensitisation. Some evidence suggests that skull and face fractures are associated with a subsequent risk of headache and migraines [43, 44]. In our study, a high correlation existed between the total ISS and ISS Face scores; thus, we included only the total ISS in our models to adjust for the overall injury severity.

Neuroimaging studies have reported that patients with PTH displayed reduced cortical thickness in various bilateral frontal and right parietal regions, with headache burden being negatively correlated to bilateral superior frontal cortex thickness [45]. Schwedt et al. [46] found that the left superior frontal lobe, right lateral orbitofrontal lobe and right supramarginal gyrus differed between persistent PTH and healthy controls, and not between migraine patients and healthy controls, suggesting a certain degree of brain structures involvement or pathophysiological specificity unique to persistent PTH, regardless of the clinical phenotype. Microvascular channels that course between the skull bone and marrow and dura mater could also be important [47]. The human skull bone marrow contains microvascular channels that allow inflammatory cells to migrate into the meninges and potentially other intracranial structures associated with headache in animal studies [47]. Most nerve fibers in the bone marrow are nociceptive. The initial mechanisms to activate meningeal nociceptors involve the release of signalling molecules like calcitonin gene-related peptide (CGRP) and pituitary adenylate cyclase-activating polypeptide-38 (PACAP-38) [48]. Both CGRP and PACAP induce migraine-like headaches in migraine patients [49]. Patients with persistent PTH may experience worsened headaches with migraine-like characteristics [50, 51]. Binding of CGRP and PACAP-38 to their G protein-coupled receptors opens ATP-sensitive potassium (K_ATP_) channels on the vascular smooth muscle cells in the walls of the meningeal arteries [48, 52, 53]. Additionally, the K_ATP_ channel opener levcromakalim induces migraine-like headaches in people with persistent PTH but no history of migraine [54]. Therefore, blocking K_ATP_ channel could be a potential drug target for PTH [54].

The presence of post-injury comorbidities (feeling depressed, fatigue, sleep disturbance, vision problems, neck pain and mobility problems) was associated with headache severity levels in the entire population in the first 6 months after the injury, and neck pain also predicted headache at 12 months in the ADM and ICU strata. Previous TBI studies with mixed-severity samples have demonstrated the association between these comorbidities and headache [27]. For example, depression and other aspects of emotional distress are common after TBI and contribute to the duration and intensity of headache [20]. Headache may also be triggered by problems in the visual system, such as vergence impairment, accommodation, fixation disparity, saccades, pursuits and secondary to oculomotor dysfunction [22]. The findings of our study are of clinical importance to rehabilitation professionals given the impact these comorbidities may have on daily activity levels, participation and health-related quality of life. Treating headache in itself and the symptoms that co-occur and interact with headache following TBI are currently the best recommendations for treatment, including a multidisciplinary approach to the clinical management of persistent headache [27].

Overall, the same factors predicted headache regardless of the applied cutoff (≥ 2 or ≥ 3), indicating the reliability of the predictors used in the study. The time since injury predicted changes on its own and interacted with a range of predictors, whereas TBI severity and neck pain appeared to be the most robust predictors in all models. Patients with TBI have significantly higher odds of sustaining a comorbid neck injury [55]. Comorbid neck pain is associated with greater headache severity and physical limitation [26] and contributes to post-concussion symptoms [56]. Major structural or other pathologies in the neck should be ruled out, and evidence-based guidelines for non-pharmacological treatment such as general exercise should be recommended [57]. Taken together, the study findings may help health professionals to identify patients at risk of persistent headache and plan individualised therapy.

#### Limitations

The study findings are not necessarily generalisable to individuals who have sustained a minimal or mild TBI without indication for a CT head scan since participants were mainly recruited from trauma referral centres. One of the major limitations of this study is the use of a single-item operationalisation of headache following TBI rather than the ICHD-3 [2]. However, the ICHD-3 does not include specific clinical features suggestive of PTH, which is a limitation of the classification system. Nevertheless, headache measured in this study represents a secondary headache attributed to injury that developed within the first two weeks after injury. In addition, this was the only opportunity to measure headache and its changes when using the CENTER-TBI data. The experience of symptoms can vary, raising the possibility that the reported ratings of headache symptoms do not reflect the overall experience (both over- and underreporting are possible). This may also be impacted by the wording of the RPQ headache item, which asks whether headache has been a problem during the previous 7 days compared to before the injury. Translating a subjective experience such as headache into an objective context is challenging. Using headache assessment instruments (including headache diaries) with established validity in specific patient groups is recommended [58]. Unfortunately, such instruments were not available in this study. Further, the usage of specific headache tools may not be as achievable in a hectic clinical setting as the broad current use of the RPQ; our results may thus be more easily transferrable to common clinical practice. However, based on the prevalence of headache after TBI found in this and several other studies, changing clinical practice towards investing more focus and time in assessing and treating headache would be beneficial for the individual patient and from a socioeconomic viewpoint.

The first assessment of headache was performed at mean 2.5 days after injury. Consequently, the proportion of patients with more severe injuries who responded to the headache question was lower at baseline than at 3, 6 and 12 months follow-up. However, to handle missing endpoint measures, logistic mixed-effect regression was applied which retain all available measures at each time point in the analysis, and gives unbiased results under the assumption of missing at random. Additionally, as we did not have a comparison group, for instance individuals who had sustained traumatic orthopedic injuries without head impact, we were unable to compare headache frequencies and establish whether the predictors were TBI-specific.

PTH has increasingly been conceptualised as a heterogeneous headache disorder, with patients often classified into sub-phenotypes that might be more responsive to specific therapies [27]. Substantial evidence suggests that a history of pre-injury migraine is one of the most consistent predictors of acute and persistent PTH across studies [39]. However, an important limitation of our study was that the history of pre-injury migraine was assessed by a single question of whether the patient reported receiving treatment for migraine before the injury, which may have led to underestimation of the presence of pre-injury migraine and a subsequent lack of association with post-injury headache. Approximately 11% of the total sample reported receiving treatment for migraine before the injury, compared to an estimated global migraine prevalence of 14% to 15% [59]. Moreover, we did not record the history of other forms of headache, such as tension-type headache.

## Conclusion

Headache is a common symptom after TBI, especially for female and younger patients. It tends to decrease within the first 3 months after injury before stabilising. A substantial proportion of patients still experience headache at 12 months after injury. Variables such as specific headache phenotypes, neurocognitive function, structural brain abnormalities and potential blood biomarkers, which were not included in this study, should be assessed in future research. Translational research is needed to advance the clinical decision-making process and targeted medical treatment of headache.

### Supplementary Information

**Additional file 1.** Headache frequency at 3, 6 and 12 months by patient stratum.

**Additional file 2.** Estimated proportion of patients with headache by GCS score up to 12 months postinjury.

**Additional file 3.** Significant interaction effects between time and predictors (RPQ cutoff >2, up to 6 months postinjury).

**Additional file 4.** Significant interaction effects between time and predictors (RPQ cutoff >3, up to 6 months postinjury).

**Additional file 5.** Significant interaction effects between time and predictors (RPQ cutoff >2, up to 12 months postinjury).

**Additional file 6.** Significant interaction effects between time and predictors (RPQ cutoff >3, up to 12 months postinjury).

** Additional file 7.** STROBE checklist.

## Data Availability

The datasets generated and/or analysed during the current study are not publicly available, but are available from the corresponding author on reasonable request.

## Abbreviations

ADM: Admitted to ward
AIS: Abbreviated injury scale
ASA-PS: American society of anesthesiologists physical status classification
CENTER-TBI: Collaborative european neurotrauma effectiveness research in traumatic brain injury
CGRP: Calcitonin gene-related peptide
CI: Confidence interval
CT: Computed tomography
ER: Emergency room
GCS: Glasgow coma scale
ICHD-3: International classification of headache disorders 3rd edition
ICU: Intensive care unit
IQR: Interquartile range
ISS: Injury severity score
PACAP-38: Pituitary adenylate cyclase-activating polypeptide-38
PTH: Posttraumatic headache
RPQ: Rivermead post concussion symptoms questionnaire
SD: Standard deviation
TBI: Traumatic brain injury
TRACK-TBI: Transforming research and clinical knowledge in TBI
