# Supplementary material for: Frequency and predictors of headache in the first 12 months after traumatic brain injury: results from CENTER-TBI
Source: J Headache Pain. 2024 Mar 25;25(1):44. doi: 10.1186/s10194-024-01751-0 (PMC10964672; doi:10.1186/s10194-024-01751-0)
Supplement: Supplementary file 1 — Additional file 1. Headache frequency at 3, 6 and 12 months by patient stratum. [file 10194_2024_1751_MOESM1_ESM.docx]

**Additional file 1.** **Headache frequency at 3, 6 and 12 months by patient stratum.**

| **3 months** | Total  (n=2162) | ER  (n=481) | ADM  (n=836) | ICU (n=845) | p-value |
| --- | --- | --- | --- | --- | --- |
| Median (IQR) |  | 0 | 1 | 1 |  |
| Headache score ≥2 | 689 (31.9%) | 144 (29.9%) | 268 (32.1%) | 277 (32.8%) | 0.559 |
| Headache score ≥3 | 338 (15.6%) | 77 (16%) | 124 (14.8%) | 137 (16.2%) | 0.714 |
| Headache score = 4 | 94 (4.3%) | 26 (5.4%) | 37 (4.4%) | 31 (3.7%) | 0.326 |
| **6 months** | Total  (n=2253) | ER  (n=466) | ADM  (n=852) | ICU  (n=935) |  |
| Median (IQR) |  | 0 | 0 | 0 |  |
| Headache score ≥2 | 663 (29.4%) | 131 (28.1%) | 247 (29%) | 285 (30.5%) | 0.617 |
| Headache score ≥3 | 327 (14.5%) | 60 (12.9%) | 125 (14.7%) | 142 (15.2%) | 0.505 |
| Headache score = 4 | 87 (3.9%) | 15 (3.2%) | 35 (4.1%) | 37 (4.0%) | 0.711 |
| **12 months** | Total  (n=1450) |  | ADM  (n=706) | ICU  (n=744) |  |
| Median (IQR) |  |  | 0 | 0 |  |
| Headache score ≥2 | 376 (25.9%) |  | 165 (23.4%) | 211 (28.4%) | 0.030 |
| Headache score ≥3 | 163 (11.2%) |  | 67 (9.5%) | 96 (12.9%) | 0.040 |
| Headache score = 4 | 36 (2.5%) |  | 15 (2.1%) | 21 (2.8%) | 0.393 |
