# Supplementary material for: Frequency and predictors of headache in the first 12 months after traumatic brain injury: results from CENTER-TBI
Source: J Headache Pain. 2024 Mar 25;25(1):44. doi: 10.1186/s10194-024-01751-0 (PMC10964672; doi:10.1186/s10194-024-01751-0)
Supplement: Supplementary file 2 — Additional file 2. Estimated proportion of patients with headache by GCS score up to 12 months postinjury. [file 10194_2024_1751_MOESM2_ESM.pdf]

Additional file 2. Estimated proportion of patients with headache by GCS score up to 12 months postinjury.

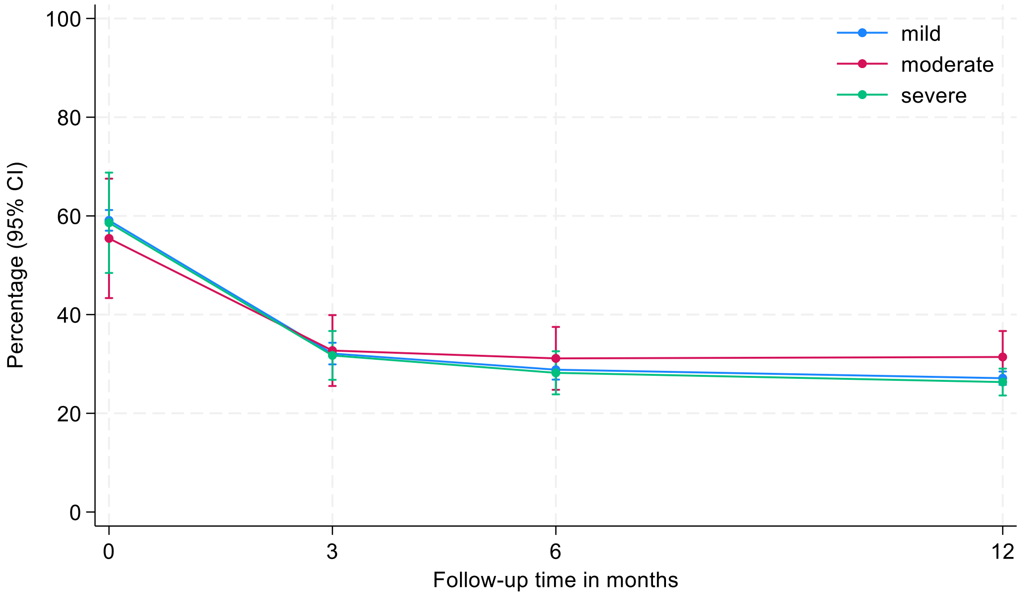

Injury severity	Baseline Percentage (95% CI)	3 months Percentage (95% CI)	6 months Percentage (95% CI)	12 months Percentage (95% CI)	Within- group difference baseline to 12 months, (95% CI)	Between group difference, baseline to 12 months, (95% CI), p-value
GCS						
Mild TBI	59.1 (57.0, 61.2)	32.1 (30.0, 34.3)	28.8 (26.8, 30.9)	27.1 (25.8, 28.5)	-32.0 (-34.4, -29.6)	Reference group
Moderate TBI	55.4 (43.4, 67.5)	32.7 (25.5, 39.9)	31.1 (24.8, 37.5)	31.4 (26.2, 36.7)	-24.0 (-37.0, -11.0)	8.0 (-5.3, 21.2), p=0.957
Severe TBI	58.6 (48.4, 68.8)	31.7 (26.8, 36.7)	28.2 (23.8, 32.5)	26.3 (23.6, 29.5)	-32.3 (-42.7, -21.9)	-0.3 (-11.0, 10.4), p=0.499

Note: Values represent estimated proportion of patients with mild (GCS score 13-15), moderate (GCS score 9-12) and severe (GCS score 3-8) TBI who reported headache (RPQ cutoff $\geq$2) at baseline, 3, 6 and 12 months follow-up.
