# Supplementary material for: Frequency and predictors of headache in the first 12 months after traumatic brain injury: results from CENTER-TBI
Source: J Headache Pain. 2024 Mar 25;25(1):44. doi: 10.1186/s10194-024-01751-0 (PMC10964672; doi:10.1186/s10194-024-01751-0)

Additional file 5. Significant interaction effects between time and predictors (RPQ cutoff $\geq$2, up to 12 months postinjury).

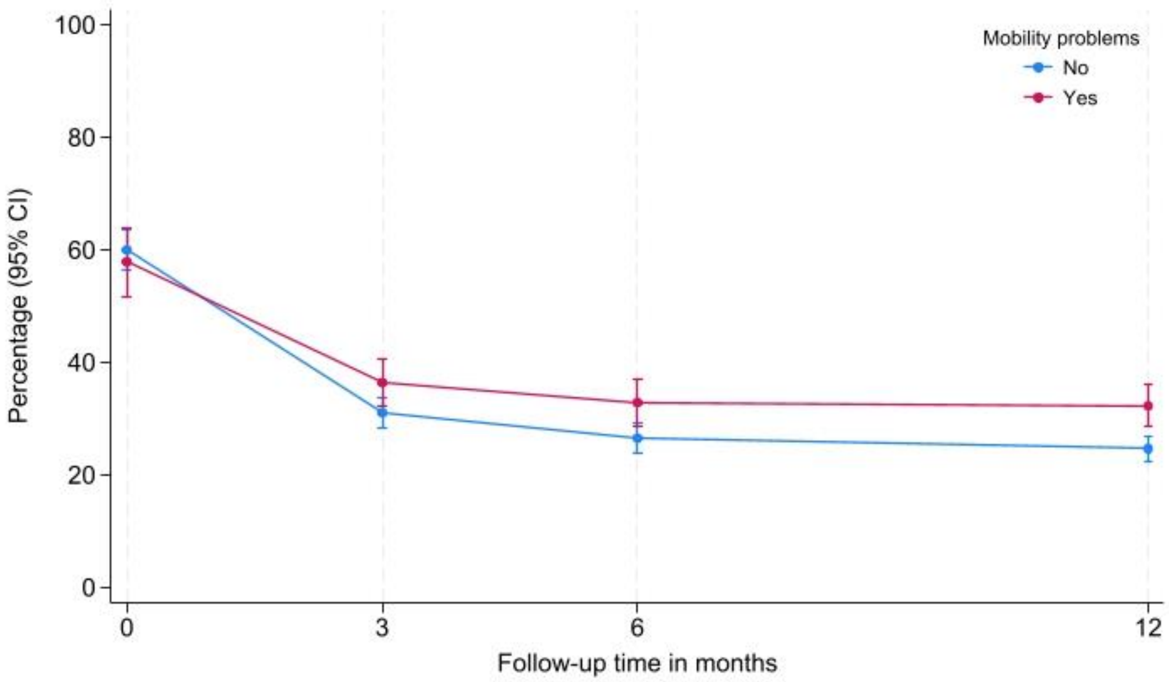

Supplement: Supplementary file 5 — Additional file 5. Significant interaction effects between time and predictors (RPQ cutoff >2, up to 12 months postinjury). [file 10194_2024_1751_MOESM5_ESM.pdf]
