# Supplementary material for: Frequency and predictors of headache in the first 12 months after traumatic brain injury: results from CENTER-TBI
Source: J Headache Pain. 2024 Mar 25;25(1):44. doi: 10.1186/s10194-024-01751-0 (PMC10964672; doi:10.1186/s10194-024-01751-0)
Supplement: Supplementary file 7 — Additional file 7. STROBE checklist. [file 10194_2024_1751_MOESM7_ESM.pdf]

Additional file 7. STROBE checklist.

STROBE Statement—Checklist of items that should be included in reports of ***cohort studies***

	Item No	Recommendation
**Title and abstract**	1	(*a*) Indicate the study's design with a commonly used term in the title or the abstract Prospective observational cohort study as stated in the abstract.
		(*b*) Provide in the abstract an informative and balanced summary of what was done and what was found Provided in the abstract.
**Introduction**		
Background/rationale	2	Explain the scientific background and rationale for the investigation being reported Included in the background section on pages 3-5.
Objectives	3	State specific objectives, including any prespecified hypotheses Specific objectives and hypothesis is included at the bottom of the background section on page 5.
**Methods**		
Study design	4	Present key elements of study design early in the paper Presented in the background section (p. 4) and under the study design subsection of the methods section (p. 5).
Setting	5	Describe the setting, locations, and relevant dates, including periods of recruitment, exposure, follow-up, and data collection Included on page 5.
Participants	6	(*a*) Give the eligibility criteria, and the sources and methods of selection of participants. Describe methods of follow-up Presented in the methods section on pages 5 and 8.
		(*b*) For matched studies, give matching criteria and number of exposed and unexposed Not applicable.
Variables	7	Clearly define all outcomes, exposures, predictors, potential confounders, and effect modifiers. Give diagnostic criteria, if applicable Included on pages 6-8.
Data sources/ measurement	8*	For each variable of interest, give sources of data and details of methods of assessment (measurement). Describe comparability of assessment methods if there is more than one group Included in the methods section on pages 7 and 8.
Bias	9	Describe any efforts to address potential sources of bias Addressed in the limitations section on pages 25-26.
Study size	10	Explain how the study size was arrived at No a priori sample size calculation has been done as the number of participants will be fixed on the 4,509 patients included in The CENTER-TBI core study.
Quantitative variables	11	Explain how quantitative variables were handled in the analyses. If applicable, describe which groupings were chosen and why Included on pages 7-8
Statistical methods	12	(*a*) Describe all statistical methods, including those used to control for confounding Included on pages 8-10
		(*b*) Describe any methods used to examine subgroups and interactions Included on pages
		(*c*) Explain how missing data were addressed Included on pages 9
		(*d*) If applicable, explain how loss to follow-up was addressed Not applicable

		(*e*) Describe any sensitivity analyses Included on pages
**Results**		
Participants	13*	(a) Report numbers of individuals at each stage of study—eg numbers potentially eligible, examined for eligibility, confirmed eligible, included in the study, completing follow-up, and analysed Included on page 6.
		(b) Give reasons for non-participation at each stage Included on page 25.
		(c) Consider use of a flow diagram Not required.
Descriptive data	14*	(a) Give characteristics of study participants (eg demographic, clinical, social) and information on exposures and potential confounders Included in table 1.
		(b) Indicate number of participants with missing data for each variable of interest
		(c) Summarise follow-up time (eg, average and total amount) Not relevant.
Outcome data	15*	Report numbers of outcome events or summary measures over time
Main results	16	(*a*) Give unadjusted estimates and, if applicable, confounder-adjusted estimates and their precision (eg, 95% confidence interval). Make clear which confounders were adjusted for and why they were included Not applicable
		(*b*) Report category boundaries when continuous variables were categorized See results section and additional files
		(*c*) If relevant, consider translating estimates of relative risk into absolute risk for a meaningful time period Not applicable
Other analyses	17	Report other analyses done—eg analyses of subgroups and interactions, and sensitivity analyses See results section and additional files
**Discussion**		
Key results	18	Summarise key results with reference to study objectives Included on page 21.
Limitations	19	Discuss limitations of the study, taking into account sources of potential bias or imprecision. Discuss both direction and magnitude of any potential bias Reported on pages 25-26.
Interpretation	20	Give a cautious overall interpretation of results considering objectives, limitations, multiplicity of analyses, results from similar studies, and other relevant evidence Included in the discussion on pages 21-24.
Generalisability	21	Discuss the generalisability (external validity) of the study results Included on page 25.
**Other information**		
Funding	22	Give the source of funding and the role of the funders for the present study and, if applicable, for the original study on which the present article is based Reported on pages 27-28.

*Give information separately for exposed and unexposed groups.

Note: An Explanation and Elaboration article discusses each checklist item and gives methodological background and published examples of transparent reporting. The STROBE checklist is best used in conjunction with this article (freely available on the Web sites of PLoS Medicine at http://www.plosmedicine.org/, Annals of Internal Medicine at

http://www.annals.org/, and Epidemiology at http://www.epidem.com/). Information on the STROBE Initiative is available at http://www.strobe-statement.org.
